# Isoprenoid Derivatives of Lysophosphatidylcholines Enhance Insulin and GLP-1 Secretion through Lipid-Binding GPCRs

**DOI:** 10.3390/ijms22115748

**Published:** 2021-05-27

**Authors:** Anna Drzazga, Daria Kamińska, Anna Gliszczyńska, Edyta Gendaszewska-Darmach

**Affiliations:** 1 Institute of Molecular and Industrial Biotechnology, Faculty of Biotechnology and Food Sciences, Lodz University of Technology, Stefanowskiego 4/10, 90-924 Lodz, Poland; anna.drzazga@p.lodz.pl (A.D.); daria.kaminska@dokt.p.lodz.pl (D.K.); 2 Department of Chemistry, Wrocław University of Environmental and Life Sciences, Norwida 25, 50-375 Wrocław, Poland

**Keywords:** diabetes, insulin, GLP-1, GPR40, GPR55, GPR119, GPR120, lysophosphatidylcholine, isoprenoids

## Abstract

Insulin plays a significant role in carbohydrate homeostasis as the blood glucose lowering hormone. Glucose-induced insulin secretion (GSIS) is augmented by glucagon-like peptide (GLP-1), a gastrointestinal peptide released in response to ingesting nutriments. The secretion of insulin and GLP-1 is mediated by the binding of nutrients to G protein-coupled receptors (GPCRs) expressed by pancreatic β-cells and enteroendocrine cells, respectively. Therefore, insulin secretagogues and incretin mimetics currently serve as antidiabetic treatments. This study demonstrates the potency of synthetic isoprenoid derivatives of lysophosphatidylcholines (LPCs) to stimulate GSIS and GLP-1 release. Murine insulinoma cell line (MIN6) and enteroendocrinal L cells (GLUTag) were incubated with LPCs bearing geranic acid (1-GA-LPC), citronellic acid (1-CA-LPC), 3,7-dimethyl-3-vinyloct-6-enoic acid (GERA-LPC), and (E)-3,7,11-trimethyl- 3-vinyldodeca-6,10-dienoic acid (1-FARA-LPC). Respective free terpene acids were also tested for comparison. Besides their insulin- and GLP-1-secreting capabilities, we also investigated the cytotoxicity of tested compounds, the ability to intracellular calcium ion mobilization, and targeted GPCRs involved in maintaining lipid and carbohydrate homeostasis. We observed the high cytotoxicity of 1-GERA-LPC and 1-FARA-LPC in contrast 1-CA-LPC and 1-GA-LPC. Moreover, 1-CA-LPC and 1-GA-LPC demonstrated the stimulatory effect on GSIS and 1-CA-LPC augmented GLP-1 secretion. Insulin and GLP-1 release appeared to be GPR40-, GPR55-, GPR119- and GPR120-dependent.

## 1. Introduction

The pancreas is a crucial organ in tight control of blood glucose levels. β-cells located within Langerhans’ islets secrete insulin, further stimulating glucose uptake into skeletal muscles and adipose tissue. However, impaired/absent insulin signaling may lead to metabolic disorders such as type 2 diabetes mellitus (T2DM), a significant public health concern due to its pandemic occurrence and several comorbidities and dramatic medical costs [1].

Blood glucose homeostasis is under the control of glucagon-like peptide-1 (GLP-1) augmenting glucose-stimulated insulin secretion (GSIS). GLP-1, a peptide secreted from intestinal enteroendocrine L cells, is also proposed to act as a factor in reducing appetite and food intake working on the central nervous system [2]. Although GLP-1 mimetics show glucose-lowering efficacies as well as notable weight-loss effects, treatment with exenatide and sitagliptin, GLP-1–derived medications, was supposed to increase cancer risk, especially pancreatic and thyroid carcinomas [3]. Cases of pancreatitis were also described in connection with the use of exenatide, liraglutide, and other GLP-1 analogs. Prescribing information and warning labels for exenatide and liraglutide list pancreatitis as a potential side effect of their use [4]. It is anticipated that an approach to increase endogenous GLP-1 secretion rather than administering exogenous GLP-1 mimetics will be popularized. Such compounds should also have a reduced propensity to promote cell growth while retaining their capacity to stimulate pancreatic insulin secretion.

Several nutrients affect cellular behavior by activation of specific GPCRs. For example, the secretion of GLP-1 is primarily mediated by the binding of many different nutrient metabolites such as short, medium, and long chain fatty acids, amino acids, and peptides to specialized GPCRs expressed by enteroendocrine gastrointestinal cells [2]. Furthermore, food-derived carbohydrates, peptides, amino acids, lipids, or alkaloids binding GPCRs are responsible for detecting sweet, bitter, and umami tastes [5]. GPCRs also control the exocytosis of insulin granules and the β-cell survival by the second messenger pathways. Almost 300 GPCRs expressed in pancreatic islets have been identified; however, most of these receptors are still considered orphans because their agonists and/or their signaling properties are currently unknown [6]. GLP-1 receptor (GLP-1R) is the most abundant GPCR in pancreatic islets, potentiating GSIS through insulin gene transcription, insulin mRNA translation, proinsulin biosynthesis, and β-cell proliferation [4]. Activation of lipid-binding GPCRs such as GPR40, GPR55, GPR119, and GPR120, is also known to potentiate GSIS from β-cells. Their prominent role in pancreatic cells and peripheral tissues biology has become a hot frontier in basic research and therapeutic discovery. GPR40 (FFA1R) is activated by medium chain fatty acids and long chain fatty acids (LCFAs), and GPR120 (FFAR4) by LCFAs. In turn, GPR119 is activated by oleoylethanolamide (OEA) and other LCFA ethanolamides, derivatives of oleic acid such as oleoyldopamine and 2-oleoyl glycerol (2-OG), lysophosphatidylcholine (LPC), and lysophosphatidylinositol. GPR55 is another GPCR target for fatty acid-derived lipids, e.g., endocannabinoids, lysophosphatidylcholine, and lysophosphatidylinositol [7].

**Figure 1:**
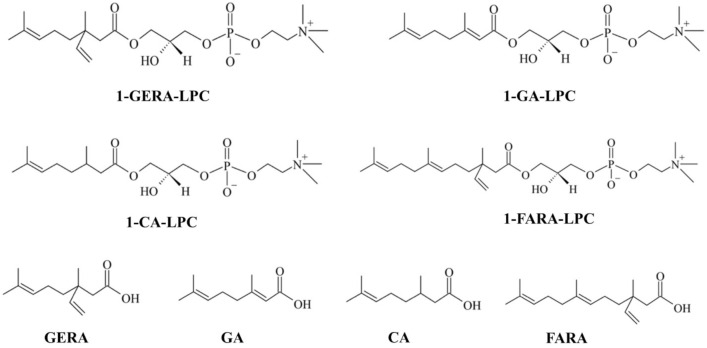
Isoprenoid derivatives of lysophosphatidylcholine: 1′-[3,7-dimethyl-3-vinylocta-6-enyl]-2′-hydroxy-sn-glycero-3′-phosphocholine (1-GERA-LPC), 1-geranoyl-2-hydroxy-sn-glycero-3-phosphocholine (1-GA-LPC), 1-citroneloyl-2-hydroxy-sn-glycero-3-phosphocholine (1-CA-LPC), 1′-[3,7,11-trimethyl-3-vinyldodec-6,10-dienyl]-2′-hydroxy-sn-glycero-3′-phosphocholine (1-FARA-LPC), free monoterpene acids: 2,3-dihydro-3-vinylgeranic acid (GERA), geranic acid (GA), cytronellic acid (CA), and free sesquiterpene acid: (E)-3,7,11-trimethyl-3-vinyldodeca-6,10-dienoic acid (FARA).

LPC is a well-known signaling molecule playing a role in various physiological processes, including modulation of insulin secretion as a ligand of various GPCR receptors [8]. Most popular LPCs contain fatty acyl residues of different chain lengths and saturation. LPC 16:0 is the most abundant species in human plasma followed by LPC 18:0 and LPC 18:1 [9]. The role of LPCs in the context of diabetes has been unraveled by a seminal experiment in which diabetic mice showed dose-dependent reduction of blood glucose levels compared to the hypoglycaemic activity of insulin [10]. What is more, lipidomic analysis revealed a correlation of lower plasma concentrations of LPCs with impaired glucose tolerance and obesity. Lower levels of LPC were also found to be predictors of T2DM [11], indicating that the proper concentration of LPC is necessary to prevent diabetes. Our previous studies indicated that in MIN6 cells LPCs with lauroyl (12:0), mirystoyl (14:0), palmitoyl (16:0), stearolyl (18:0), and oleoyl (18:1) fatty acid residues significantly potentiated the level of GSIS above the control level (from 1.5 to 3.5-fold). In this case, LPCs bearing longer acyl chains tended to stimulate MIN6 cells stronger than those bearing medium acyl chains [12]. Additionally, we demonstrated that LPC bearing oleoyl and palmitoyl fatty acid residues augmented GSIS by targeting GPR40, GPR55, and GPR119 receptors, which represent a novel antidiabetic approach [12,13]. Moreover, we identified new insulin secretion modulators synthesized by combining the structures of two natural compounds, namely *O*-methyl derivatives of phenolic acids and LPC. Our results showed that LPC with a covalently bonded molecule of *p*-anisic acid at the *sn*-1 position could induce GSIS and intracellular calcium flux by activating GPR40, GPR55, and GPR119 [14]. Here we show the antidiabetic activity of LPC hybrid molecules bearing various isoprenoids in the position *sn*-1, namely geranic acid (1-GA-LPC), citronellic acid (1-CA-LPC), 3,7-dimethyl-3-vinyloct-6-enoic acid (GERA-LPC), and (*E*)-3,7,11-trimethyl-3-vinyldodeca-6,10-dienoic acid (1-FARA-LPC) (Figure 1).

Isoprenoids, also known as terpenes, are the most abundant and structurally diverse group of chemical compounds found in living organisms. The growing interest in terpenes research is related to their potential clinical application. Terpenes (including natural and synthetic acyclic monoterpenes and sesquiterpenes derivatives) exhibit several biological properties that may be effective for the treatment of a variety of diseases, including antibacterial, antifungal, antiviral, antitumor, antiparasitic, hypoglycemic, anti-inflammatory, and analgesic, as well as antioxidant properties [15]. Moreover, these compounds are well known as agents that play a significant role in the posttranslational modification of proteins that are involved in regulating a wide range of cellular processes and functions and in the pathogenesis of different types of diseases [16], including T2DM [17]. All of this makes isoprenoids attractive therapeutic targets for combating various disorders.

## 2. Results

### 2.1. Determination of MIN6 Metabolic Activity and Proliferation under the Influence of Isoprenoid Derivatives of LPCs

**Figure 2:**
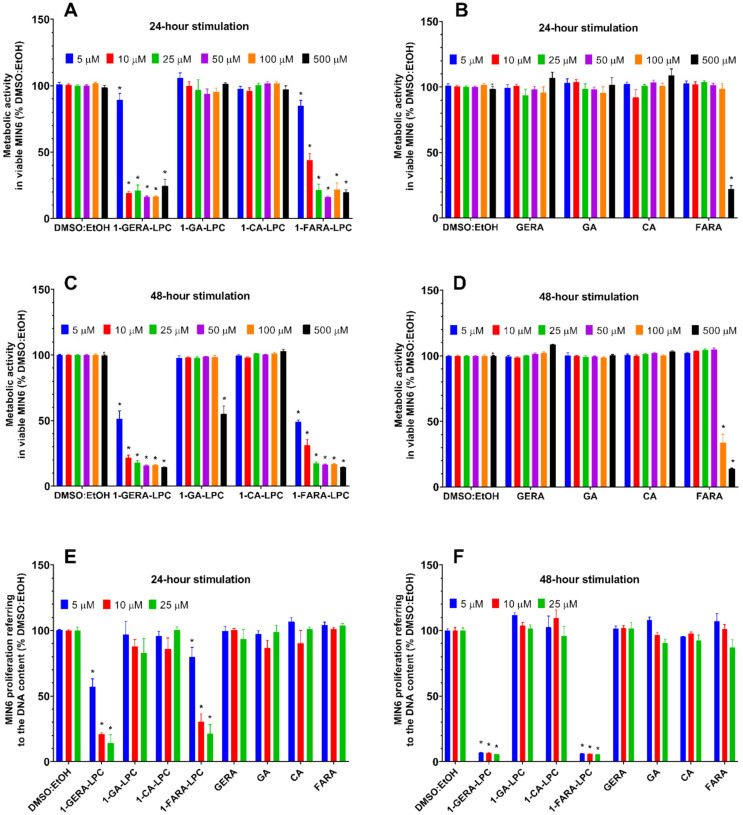
MIN6 cells viability after 24-h (**A**,**B**,**E**) and 48-h (**C**,**D**,**F**) treatment with 1-GERA-LPC, 1-GA-LPC, 1-CA-LPC, 1-FARA-LPC and corresponding free terpene acids (GERA, GA, CA, FARA). Cytotoxicity was assessed based on the activity of mitochondrial reductases with the PrestoBlue^TM^ Cell Viability Reagent (**A**–**D**) in the range of 5–500 μM concentrations (**A**–**D**). The lowest compound concentrations (5–25 μM) were re-assessed in cytotoxicity experiments referring to the number of living cells to the quantity of DNA with the CyQUANT^TM^ Cell Proliferation Test (**E**,**F**). The results are expressed as % of living cells after treatment with respective compounds solvent (DMSO:EtOH) and presented as means ± SEM from 3-4 independent experiments, 3-6 biological replicates each. * *p* < 0.05 is considered as significantly different from DMSO:EtOH.

The effect of isoprenoid derivatives of LPCs on MIN6 cells growth was determined with the rezasurin-based PrestoBlue^TM^ Cell Viability Reagent and a green fluorescent nucleic acid stain present in the CyQUANT^TM^ Cell Proliferation Test. The potential cytotoxicity of isoprenoid lysophosphatidylcholine analogs was evaluated in the concentration range of 5–500 µM after 24 and 48 h of incubation with the cells. The possible background fluorescence of tested compounds incubated solely with culture media showed no statistically significant differences compared to control media (data not shown). Results showed that after 24 h of incubation no decrease in metabolic activity was observed for 1-CA-LPC over the full range of analyzed concentrations. 1-GA-LPC reduced the viability of MIN6 cells only at the highest concentration (500 μM). The remaining analogs turned out to be highly cytotoxic to the MIN6 cell line. 1-GERA-LPC decreased the activity of MIN6 cells by about 50%, even at the lowest concentration used (5 μM) while in the case of 1-FARA-LPC, a similar effect was observed at a concentration of 10 µM (Figure 2A). All free terpene acids, except for FARA, did not affect cellular viability (Figure 2B). After 48 h exposure, high cytotoxicity was also observed for 1-GERA-LPC and 1-FARA-LPC. The reduced viability was observed at the highest concentration of 1-GA-LPC whereas 1-CA-LPC did not influence cell viability negatively (Figure 2C,D).

The influence of isoprenoid derivatives of LPCs on MIN6 proliferation measured as the DNA content was then assessed using the three lowest concentrations after 24 h and 48 h exposure. Since cytotoxicity tests based on metabolic activity measurement may not accurately reflect the actual changes in the number of cells, the commercially available CyQUANT^TM^ assay was performed. The results confirmed the highest cytotoxic effect observed for 1-GERA-LPC and 1-FARA-LPC. The most prominent reduction of DNA content by these compounds was observed after 48 h of incubation. Lack of toxicity was observed in the case 1-GA-LPC, 1-CA-LPC, and all free terpene acids (Figure 2E,F).

### 2.2. The Influence of Isoprenoid Derivatives of LPCs on GSIS and Intracellular Ca^2+^ Mobilization in MIN6 Cells

**Figure 3:**
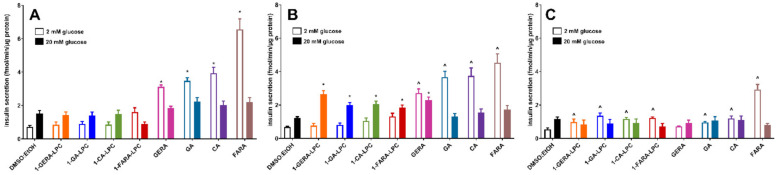
GSIS in MIN6 cells stimulated with 1-GERA-LPC, 1-GA-LPC, 1-CA-LPC, 1-FARA-LPC, and corresponding acids (GERA, GA, CA, FARA) at 5, 10 and 25 μM concentrations (**A**–**C**, respectively) versus compound solvent control (DMSO:EtOH). Results are presented as means ± SEM from 6 independent experiments, 4 biological replicates each. Secreted insulin was quantified at 2 mM glucose (open bars) and 20 mM glucose conditions (closed bars). *p* < 0.05 for secretion was significantly different from 2 mM glucose control (^) or 20 mM glucose control (*).

The three lowest concentrations (5 µM, 10 µM, and 25 µM) of LPC derivatives were used to evaluate their ability to augment *insulin secretion* from MIN6 cells under 2 mM and 20 mM glucose conditions. In control cells, high glucose concentration caused an approx. 2-fold increase in the amount of insulin released. All free terpene acids showed a statistically significant unfavorable increase of insulin secretion in low glucose conditions, even when applied at 5 μM concentration. A similar effect was observed in the case of 5 μM 1-FARA-LPC, however, the latter was not statistically significant (Figure 3A). In contrast, when used at 10 μM concentration, all LPC derivatives significantly stimulated GSIS in the presence of 20 mM glucose. Notably, only a slight increase in insulin secretion was detected upon stimulation under low glucose conditions (Figure 3B). A dose–response experiment showed that all isoprenoid derivatives of LPCs applied at a concentration of 25 μM caused an unfavorable increase in insulin secretion at 2 mM glucose concentration (Figure 3C).

In pancreatic β-cells, an increase in intracellular Ca^2^^+^ concentration ([Ca^2^^+^]_i_) is caused either by an influx of the ion through voltage-dependent or other channels in the plasma membrane or by GPCR-dependant inositol-1,4,5-trisphosphate-triggered release from the endoplasmic reticulum store is the primary trigger for GSIS [18]. Changes in [Ca^2^^+^]_i_ under the influence of isoprenoid analogs of LPC and terpene acids were studied with the Fluo-8 calcium probe’s fluorescence intensity. In MIN6 cells, intracellular calcium mobilization was controlled for 3 min after stimulation in a buffer containing 2 mM and 20 mM glucose. A concentration of 10 µM of tested compounds was selected based on the results obtained from previous experiments (cytotoxicity analysis and GSIS). To analyze cell membrane permeability during the exposure to the tested compounds, propidium iodide staining was monitored simultaneously with the calcium flux kinetics [19]. The AUC (area under the curve) parameter was used to estimate the total amount of mobilized calcium.

**Figure 4:**
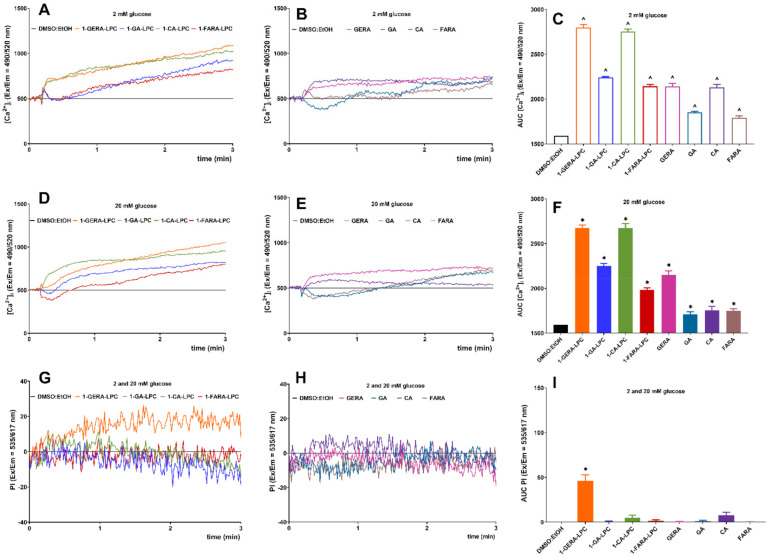
Intracellular Ca^2+^ mobilization in MIN6 cells triggered by 1-GERA-LPC, 1-GA-LPC, 1-CA-LPC, 1-FARA-LPC and corresponding acids (GERA, GA, CA, FARA) versus the compound solvent (DMSO:EtOH) at 2 mM (**A**–**C**) and 20 mM glucose conditions (**D**–**F**), with simultaneous monitoring of membrane integrity via PI incorporation (**G**–**I**)—data were collected from both 2 and 20 mM glucose conditions). The results are presented as the real-time kinetics of [Ca^2+^]_i_ and [PI] changes inside the cell during 3 min-time monitoring as well as AUC ± SEM from 3-4 independent experiments, 3-5 biological replicates each. The compounds were tested at 10 μM concentration. *p* < 0.05 is considered as significantly different from DMSO:EtOH at 2 mM (^) or 20 mM (*) glucose conditions.

The increase in the calculated AUC was statistically significant for all tested compounds at low glucose concentrations. 1-GERA-LPC and 1-CA-LPC and GERA and CA contributed to rapidly growing [Ca^2+^]_i_ immediately after stimulation which further caused a stable mobilization. The kinetics of [Ca^2+^]_i_ flux under the influence of 1-GA-LPC and 1-FARA-LPC was slightly different. After a short and sharp increase in [Ca^2^^+^]_i_ a decrease was observed, followed by a slow and stable increase throughout the monitoring period. 1-GERA-LPC and 1-CA-LPC were the most potent stimulators in low and high glucose conditions (Figure 4A–F). Importantly, simultaneous propidium iodide staining showed that 1-GERA-LPC caused a significant membrane perforation (Figure 4G–I).

### 2.3. The Role of GPR40, GPR55, GPR119, and GPR120 in GSIS and Intracellular Ca^2+^ Mobilization Evoked by Isoprenoid Derivatives of LPCs in MIN6 Cells

LPC was identified as a ligand for GPR40, GPR55, and GPR119 [13]. Our observation that isoprenoid derivatives of LPCs potently induced secretion of insulin from MIN6 cells raised the question of whether GPCRs were involved in this process. GPR40 is activated by medium and long chain fatty acids. Since similar ligands (LCFAs) also target GPR120 [7], we included the latter in the analysis. Previously, we confirmed GPR40, GPR55, and GPR119 expression in a MIN6 cell line [12], and the expression of GPR120 was also identified in this cell line [20].

Among the entire set of analyzed compounds, 1-GA-LPC and 1-CA-LPC exhibited the desired features. These compounds, applied at a concentration of 10 µM, were not cytotoxic and they stimulated insulin secretion only in high glucose as well as induced mobilization of [Ca^2+^]_i_. Therefore, 1-GA-LPC and 1-CA-LPC were selected to evaluate their ability to activate GPR40, GPR55, GPR119, and GPR120 receptors. To investigate this, we applied selective antagonists (DC, CID, C8, and AH of GPR40, GPR55, GPR119, and GPR120, respectively) to assess insulin secretion and intracellular Ca^2+^ mobilization.

**Figure 5:**
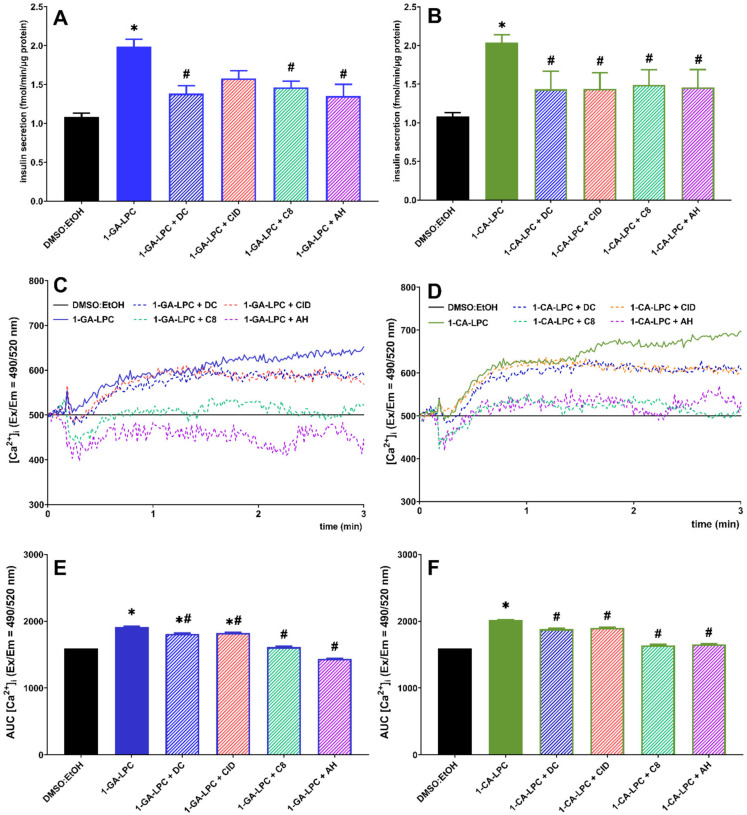
Insulin secretion (**A**,**B**) and intracellular Ca^2+^ mobilization (**C**–**F**) in MIN6 at 20 mM glucose conditions triggered by 1-GA-LPC (**A**,**C**,**E**) and 1-CA-LPC (**B**,**D**,**F**) against GPR40, GPR55, GPR119, and GPR120 antagonists (depicted as DC, CID, C8, and AH respectively). The results are presented as means ± SEM from 4 independent experiments, 4-6 biological replicates each. *p* < 0.05 was estimated as significantly different from DMSO:EtOH control (*) and from MIN6 cells untreated with receptor antagonists (^#^).

Insulin secretion induced by 1-GA-LPC and 1-CA-LPC was lowered in the presence of all antagonists. Statistically significant differences in 1-GA-LPC-induced GSIS were observed between control cells and cells incubated with antagonists of GPR40, GPR119, and GPR120 receptors (Figure 5A). In turn, treatment of cells with all antagonists of the studied GPCRs affected 1-CA-LPC-induced GSIS, reaching statistical significance (Figure 5B). We next determined whether GPR40, GPR55, GPR119, and GPR120 receptors were required for intracellular calcium mobilization evoked by 1-CA-LPC and 1-GA-LPC. Consistent with GSIS results, the increase in [Ca^2^^+^]_i_ upon MIN6 cell stimulation with 1-CA-LPC and 1-GA-LPC was reduced in the presence of all antagonists (Figure 5C–F). Co-incubation with GPR119 and GPR120 antagonists resulted in the highest inhibition. The effect of antagonists was not as high as in the case of GSIS, because insulin secretion is the final cellular response triggered by many factors. Intracellular calcium mobilization is only one of such factors. All GPCRs respond to the presence of ligands with biased agonism. Agonists of GPR40, GPR55, GPR119, and GPR120 may result in the activation of various G proteins, namely G_q/11_, G_s_, Gi_/o_ protein, and beta-arrestin-mediated signaling, all associated with GSIS [7]. Here we have investigated one of the possible intracellular signaling pathways associated with calcium ions. However, the effect observed confirms that those GPCR are activated by compounds studied.

### 2.4. The Influence of Isoprenoid Derivatives of LPCs on GLP-1 Secretion in GLUTag Cells

**Figure 6:**
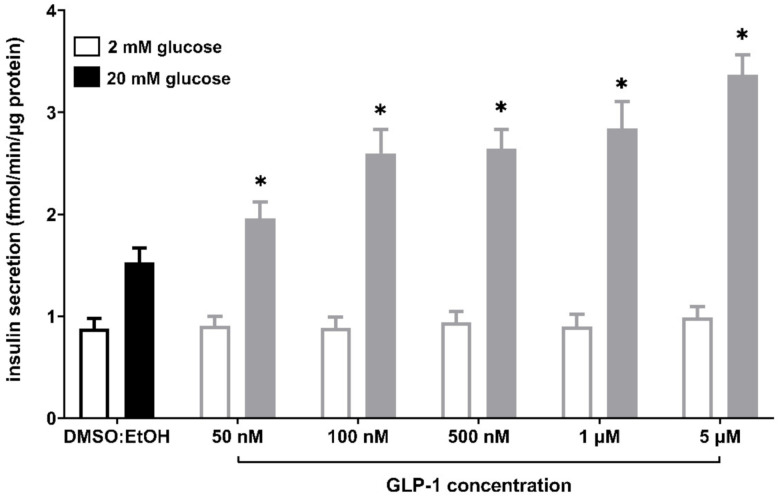
GSIS in MIN6 cells stimulated with GLP-1 in 50 nM, 100 nM, 500 nM, 1 μM, and 5 μM concentrations versus compound solvent control (DMSO:EtOH). Results are presented as means ± SEM from 3-4 independent experiments, 4-5 biological replicates each. Secreted insulin was quantified at 2 mM glucose (open bars) and 20 mM glucose conditions (closed bars). *p* < 0.05 for secretion was significantly different from 20 mM glucose control (*). No statistically significant difference was observed between control conditions and GLP-1-stimulated samples at 2 mM glucose.

Secretion of GLP-1 by enteroendocrine L cells plays a vital role in stimulating insulin secretion. Enhanced production of GLP-1 potentiates the secretion of insulin in a dose-dependent manner [21,22,23]. We performed additional GSIS experiments in the MIN6 cell line stimulated with various doses of GLP-1 to verify its influence on insulin secretion (Figure 6). The applied concentrations of GLP-1 were following standard protocols (up to 100 nM [21,22,23]) as well as the micromolar range referring to the concentration of the studied LPCs. The experiment showed that GLP-1 potentiates GSIS in MIN6 in a dose-dependent manner in high (20 mM) glucose conditions and without any significant increase in insulin production at low (2 mM) glucose conditions.

**Figure 7:**
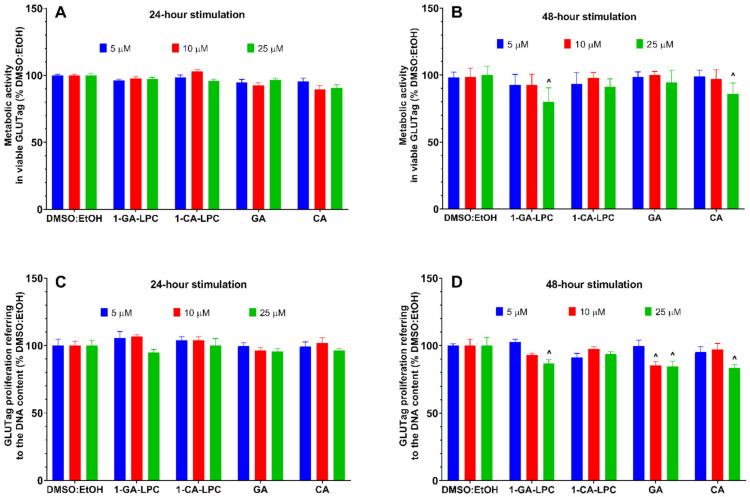
GLUTag cells viability after 24 h (**A**,**C**) and 48 h (**B**,**D**) treatment in fasting conditions with 1-GA-LPC and 1-CA-LPC and their corresponding acids (GA, CA). Cytotoxicity was assessed in the range of 5–25 μM concentrations, based on the activity of mitochondrial reductases with the PrestoBlue^TM^ Cell Viability Reagent (**A**,**B**), and referring the number of living cells to the quantity of DNA with the CyQUANT^TM^ Cell Proliferation Test (**C**,**D**). The results are presented as means ± SEM from 3-4 independent experiments, 3-6 biological replicates each, and expressed as % of living cells after treatment with respective compounds solvent (DMSO:EtOH). ^ *p* < 0.05 is considered as significantly different from DMSO:EtOH.

Having the insulinotropic properties of GLP-1 confirmed in the MIN6 cell model of β-pancreatic cells, we decided to further investigate the effect of 1-CA-LPC and 1-GA-LPC on GLP-1 release by murine GLUTag L-cells. First, the metabolic activity and proliferation of GLUTag cells were determined in the presence of 1-CA-LPC and 1-GA-LPC applied at 5 µM, 10 µM, and 25 µM concentrations. After 24 and 48 h of stimulation, no cytotoxic effect was observed in the case of 1-CA-LPC (Figure 7). 1-GA-LPC and CA presented slight cytotoxicity towards GLUTag cells when applied at 25 µM (Figure 7B,D). GA inhibited cell proliferation after 48 h when applied at 10 and 25 μM concentrations by ca. 10 % as compared to control (Figure 7D), however, no significant difference was noticed in terms of metabolic activity of GLUTag (Figure 7B).

**Figure 8:**
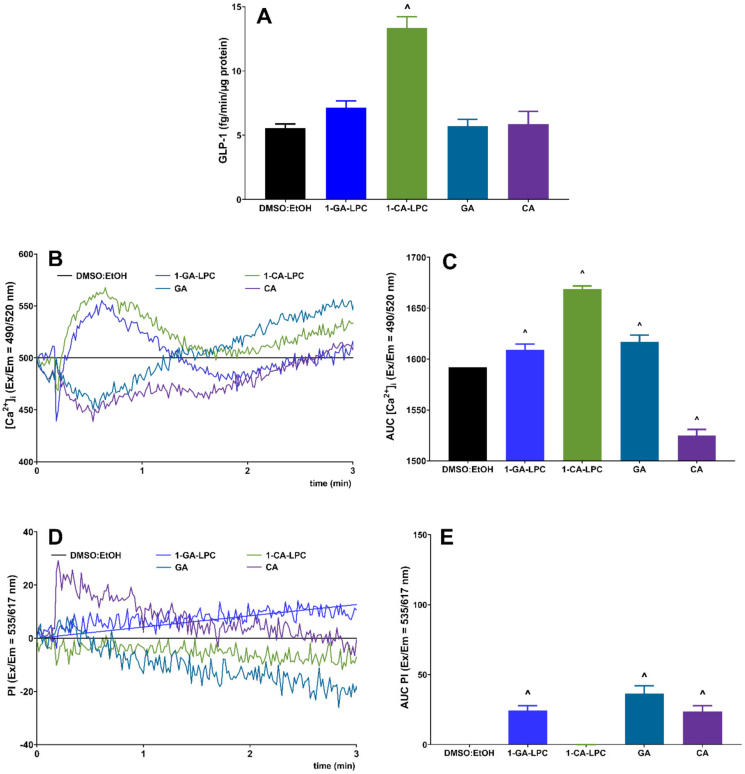
GLP-1 secretion (**A**) and intracellular Ca^2+^ mobilization (**B**,**C**) with simultaneous monitoring of membrane integrity via PI incorporation (**D**,**E**) in GLUTag cells stimulated with 10 μM 1-GA-LPC, 1-CA-LPC and their corresponding acids (GA, CA). For Ca^2+^ mobilization, the results are presented as the real-time kinetics of [Ca^2+^]_i_ and [PI] changes inside the cell during 3 min-time monitoring as well as AUC ± SEM from −4 independent experiments, 4-6 biological replicates each. *p* < 0.05 for GLP-1 secretion and Ca^2+^ mobilization was considered significantly different from EtOH:DMSO control (^).

In a subsequent series of experiments, GLP-1 responses to 1-CA-LPC and 1-GA-LPC were studied in GLUTag cells. The model of enteroendocrine L cells responded with a statistically significant increase in GLP-1 secretion when stimulated with 1-CA-LPC. Data indicated that 1-GA-LPC had a negligible effect on GLP-1 secretion when used at the same concentration as 1-CA-LPC (10 μM), whereas free acids (GA and CA) did not affect GLP-1 production at all (Figure 8A). After stimulation, 1-CA-LPC, 1-GA-LPC, and GA caused an [Ca^2+^]_i_ increase while CA reduced the amount of [Ca^2+^]_i_ below the control level (Figure 8B,C). 1-GA-LPC and GA caused membrane perforation, the increase in [Ca^2^^+^]_i_ but did not affect GLP-1 secretion. Therefore, one can speculate that the observed rise in intracellular calcium level may occur due to membrane perforation and Ca^2+^ influx from the extracellular environment. In the case of GPCR activation without membrane perforation, an increase in [Ca^2^^+^]_i_, occur as a result of its release from the intracellular Ca^2+^ stores, mainly from the lumen of the endoplasmic reticulum, where the Ca^2+^ concentration is around 1000 times higher than in the cytoplasm [24]. In turn, CA, apart from membrane perforation, is likely to destabilize calcium homeostasis by additional induction of the flux outside the cell. However, considering the insignificant influence on GLP-1 release and additional membrane perforation caused by 1-GA-LPC (Figure 8D,E), only 1-CA-LPC was chosen to investigate whether GPR40, GPR55, GPR119, and GPR120 receptors were required for 1-CA-LPC -stimulated GLP-1 release. To this end, GLUTag cells were incubated with selective antagonists before stimulation with 1-CA-LPC, since the expression of GPCRs studied hereby was confirmed previously [25,26,27].

**Figure 9:**
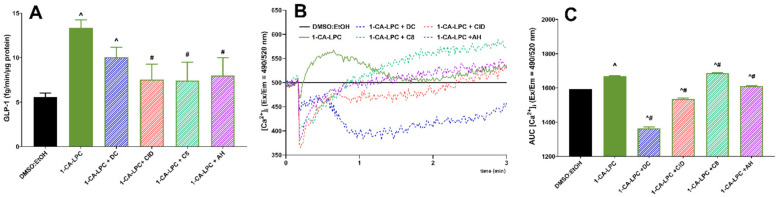
GLP-1 secretion (**A**) and intracellular Ca^2+^ mobilization (**B**,**C**) in GLUTag cells evoked by 1-CA-LPC against GPR40, GPR55, GPR119, and GPR120 antagonists (depicted as DC, CID, C8, and AH respectively). The results are presented as means (± SEM) from 4–6 independent experiments, 3–6 biological replicates each, *p* < 0.05 for GLP-1 secretion and Ca^2+^ mobilization was considered as significantly different from DMSO:EtOH control (^) and from GLUTag cells untreated with receptor antagonists (^#^).

Application of receptor antagonists led to a decrease in GLP-1 secretion from GLUTag cells, however, only in the case of GPR55, GPR119, and GPR120, the obtained results were statistically significant (Figure 9A). Antagonists affected the kinetics of [Ca^2+^]_i_ flux evoked by 1-CA-LPC, leading to a sudden drop in [Ca^2+^]_i_ shortly after the addition of the compound (Figure 9B). Comparing secretion and calcium mobilization studies, it may be suspected that weaker [Ca^2+^]_i_ flux at the beginning of the stimulation entails lowered GLP-1 secretion. In particular, the GPR40 antagonist (DC) application caused an insignificant decrease in GLP-1 secretion evoked by 1-CA-LPC by 20%, which corresponds to the most significant reduction in total [Ca^2+^]_i_ by a similar portion. Contrary to the remaining antagonist’s understudy, DC caused a milder drop in the initial [Ca^2+^]_i_ prior to further decreasing the calcium signal. Antagonists of GPR55, GPR119, and GPR120 caused the most substantial drop in GLP-1 secretion as well as in the initial [Ca^2+^]_i_ flux followed by a further increase in the calcium signal (Figure 9B).

## 3. Discussion

Although there is a lot of research into monoterpenes’ anticancer, antimicrobial, and antioxidant properties, little is known about their impact on the development of type 2 diabetes. However, the results of studies carried out in the in vitro models towards pancreatic β-cells, muscles, adipocytes, and liver cells are very promising [28,29,30]. As one of a few examples, limonene was shown to stimulate glucose uptake in 3T3-L1 adipocytes and C2C12 cells muscle cells and prevent insulin resistance [31,32]. Limonene also exhibited an inhibitory effect on α-glucosidase activity. However, the inhibitory effect was reported at 10 mM concentration, with no therapeutic relevance [32]. Good effects of isoprenoids were also reported in the in vivo antidiabetic models including the streptozotocin (STZ)-induced diabetic, high-fat diet (HFD) fed, and spontaneously obese animal models [33,34,35]. Oral administration of citronellol [33] and geraniol [34] increased the levels of insulin, hemoglobin (Hb), and hepatic glycogen with a significant decrease in glucose and glycated hemoglobin (HbA1C). The altered activities of carbohydrate metabolic enzymes, hepatic and kidney markers were restored to near normal. Citronellol and geraniol were also found to be effective in preserving the normal histological appearance of hepatic cells and insulin-positive β-cells in STZ-rats. In turn, limonene significantly decreased the oxidative stress parameters such as superoxide dismutase, catalase, glutathione reductase, and glutathione peroxidase activities. The blood glucose levels, total cholesterol, and triglyceride levels in the limonene treated diabetic rats were lower than in the control group [35]. Limonene treatment also reversed the changes in plasma HbA1C levels, the activities of gluconeogenic enzymes such as glucose 6-phosphatase and fructose 1,6-bisphosphatase, and the glucokinase activity [36].

Geranic and citronellic acids also showed antihyperglycemic activity in STZ-induced diabetic mice. GA showed a reduction in postprandial glucose peaks after a glucose load in an oral glucose tolerance test. In turn, CA decreased postprandial glucose peaks after a sucrose or lactose load in the oral sucrose and lactose tolerance tests. With these results, in the cases of CA, **the authors** suggest that reducing the postprandial peaks of glucose is mediated by the inhibition of α-glucosidase in the small intestine. In the case of GA, it is suggested that this compound acts selectively on the sodium-glucose type 1 (SGLT-1) cotransporter. The results indicate that geranic and citronellic acids are good candidates for developing and searching for new targeted treatments of diabetes mellitus [15].

However, it should be noted that the low oral bioavailability and solubility of isoprenoids may limit the potential therapeutic efficacy despite promising in vitro and in vivo results. This is the consequence of their high lipophilicity and the relatively low plasma levels may not reflect tissue distribution and concentration. One strategy that might be of better utility than using the isoprenoids as single agents is to combine them with other compounds. An especially promising strategy is the covalent binding of isoprenoids with lipids. This solution enables a release of the active substance only as a result of the action of endogenous enzymes present in the target tissues. Clinical studies have shown that therapeutic compounds administered in the form of Lipid Drug Conjugate (LDC) are characterized by higher oral bioavailability and lower toxicity in relation to free drug forms. Moreover, drug release from such combinations can be controlled, limiting the occurrence of potential side effects [37]. In recent years, the Food and Drug Administration (FDA) and the European Medicines Agency have approved the first drugs produced in the form of lipid conjugates, which have been used especially in the treatment of diabetes, schizophrenia, and depression [38].

Our previous papers reported that phospholipid conjugates with isoprenoid molecules and proved their higher antiproliferative activity towards selected cancer cell lines. Moreover, it was confirmed that at the established active concentrations they are characterized by the selective action against neoplastic cells, remaining non-toxic to cells with an undisturbed proliferation process [39,40]. Therefore, in the current study, the conjugates of lysophosphatidylcholine with geranic acid, cytronellic acid, 2,3-dihydro-3-vinylgeranic acid, and (*E*)-3,7,11-trimethyl-3-vinyldodeca-6,10-dienoic acid were applied. We synthesized a series of isoprenoid lysophosphatidylcholines to better understand the effect of conjugation of LPC with isoprenoid acids on the antidiabetic activity and try to extend the application of isoprenoids as health-promoting molecules. We used isoprenoid acids from the group of monoterpenoids and sesquiterpenoids as acyl donors, trying to find significant structure-activity relationships between the length of the chain and its degree of unsaturation and their activity towards insulin and GLP-1 secretion. We checked the stability of isoprenoid lysophospholipids in storage tests (data not shown) and it is comparable to stability of lysophospholipids containing fatty acids.

1-CA-LPC and 1-GA-LPC were previously tested for their antiproliferative activity towards human cancer cell lines, such as MV4-11 (human leukemia), A549 (lung cancer), MCF-7 (breast cancer), HepG2 (liver cancer), LoVo (colon cancer), and BALB/3T3 (normal murine fibroblasts). Their IC_50_ values ranged between 246.2 and 551.9 μM [40]. On the other hand, our previous research on unmodified LPCs and methoxy or phosphorothioate analogs of LPCs demonstrated that in the case of 10-µM concentration of natural and modified LPC species there was no significant effect on the viability of the β-cell model, yet, 25 µM appeared to be toxic [13,41,42]. Therefore, based on our previous results, concentrations ranging from 5 µM to 500 µM were chosen to determine nontoxic doses toward the MIN6 cell line for further studies. Both assays applied revealed the high cytotoxicity of 1-GERA-LPC and 1-FARA-LPC. In contrast, the reduced viability was observed at the highest concentration of 1-GA-LPC 1-CA-LPC did not affect the cell viability. No cytotoxic effect of 1-CA-LPC and 1-GA-LPC was also observed in GLUTag cells. A slight decrease of GLUTag metabolic activity and DNA content was observed only for 1-GA-LPC applied at 25 µM concentration.

As an anabolic hormone, insulin controls the metabolism of carbohydrates, lipids, and protein. Insulin promotes energy storage in the fasting state and energy utilization and uptake in the fed state. In so doing, it maintains serum glucose levels within a narrow physiologic range despite variation in energy intake and expenditure. Therefore, GLP-1, for example, is released from intestinal cells in response to the presence of food in the gut, and like other incretins, it potentiates insulin secretion at high, but not at low glucose [43]. Stimulants that evoke insulin secretion even at low level may contribute to inducing peripheral insulin resistance. Elevated basal insulin secretion under fasting conditions along with deficient stimulated insulin secretion is an important indication of type 2 diabetes [44]. Experiments with MIN6 cell line showed that *all LPC derivatives studied* (1-GA-LPC, 1-CA-LPC, 1-GERA-LPC, and 1-FARA-LPC) significantly stimulated GSIS when used at *10 μM concentration*. Importantly, no or negligible increase in insulin secretion was detected upon stimulation in low glucose conditions. The dose response experiment showed that all isoprenoid derivatives of LPCs applied at a concentration of 25 μM caused an unfavorable increase in insulin secretion at low glucose. We applied a standard GSIS protocol where cells are exposed to a low glucose concentration prior to glucose. In humans, plasma insulin peak occurs at 30 min during normal glucose response [45], and such a time point was used in our studies. *All free terpene acids showed an* unfavorable *increase of insulin secretion* in low glucose, demonstrating that lipophilization with LPC backbone is necessary for proper insulin secretion. The increased insulin secretion under low glucose level (2 mM) was particularly noticeable in the case of all free terpene acid used at all concentrations (5 µM, 10 µM, and 25 µM). Under high glucose (20 mM), the insulin release was significantly lower or even comparable to the control. Such an effect was not observed in the case of LPC derivatives of terpene acid. At a concentration of 25 μM insulin secretion was depleted at low and high glucose conditions, resulting from too high concentrations of compound applied and an adverse effect on cellular health. We can speculate that one reason for reduced GSIS in the case of free terpene acids is the strong antioxidant activity of phenolic acids [46]. There are examples where antioxidant activities depend strictly on the level of glucose. A very similar effect to that observed for free terpene acids was demonstrated for geniposide, a well-known iridoid glycoside compound and an essential component of a wide variety of traditional phytomedicines. Geniposide exerts antidiabetic activity acting as an agonist of glucagon-like peptide receptor. However, in INS-1 pancreatic cells, geniposide increased insulin secretion in the presence of low (5 mM) and moderate (11 mM) concentrations of glucose but decreased in presence of high glucose (25 mM). Additional observation revealed that when INS-1 cells were incubated with 5, 11, and 25 mM glucose, the level of intracellular H_2_O_2_ was increased in a dose-dependent manner, and geniposide augmented the accumulation of H_2_O_2_ in the presence of low and moderate concentrations of glucose. At 25 mM glucose, geniposide attenuated the level of H_2_O_2_. Those data suggest that geniposide-regulating GSIS might be associated with its role on the redox balance in pancreatic β cells [47]. A similar situation might occur in our studies, but further studies are needed to confirm this hypothesis.

Further experiments demonstrated that the stimulatory effect of 1-GA-LPC and 1-CA-LPC on insulin release and Ca^2+^ signaling in MIN6 cells was GPR40-, GPR55-, GPR119- and GPR120-dependent After ligand stimulation, GPCRs undergo a conformational change and activate the intracellular G-proteins, which are composed of α, β, and γ subunits, and then initiate signaling to the cell interior. In the G_αs_ pathway, *α_s_* subunit of G-protein activates adenylyl cyclase and in the G_αi/o_ pathway, *α_i_*_/*o*_ subunit inhibits cAMP production. In turn, in the G_αq_ pathway the *α_q_* subunit is activated, which further stimulates phospholipase C to catalyze the conversion of a phosphatidylinositol 4,5-bisphosphate (PIP_2_) into IP_3_ and diacylglycerol (DAG). IP_3_ binds to IP_3_-receptor on the endoplasmic reticulum surface (ER), leading to the transportation of Ca^2+^ from ER into the cytoplasm [48]. Mobilization of intracellular calcium stores following GPCR activation is critical for cells to respond to intercellular and environmental cues. In β-cells, the elevation of the calcium concentration in the cytosol triggers insulin secretion [49]. In the case of the studied GPCRs, GPR40, GPR55, and GPR120 couple predominantly with G_αq_ family [7]. The main signaling pathway linked to GPR119 is related to G_αs_ [35], however, we revealed that phosphorothioate analogs of LPCs did not lead to a significant cAMP increase but stimulated intracellular Ca^2+^ via GPR119-dependent manner [7,13].

We also show that 1-CA-LPC, but not 1-GA-LPC, mediates GLP-1 secretion from GLUTag cells through GPR55, GPR1119, and GPR120 as this activity was ablated in the presence of respective antagonists. Our studies demonstrate that GPR40 plays only a *minor* role in GLUTag activation by 1-CA-LPC. Given that elevation of cytosolic Ca^2+^ is a crucial requirement for GLP-1 secretion in GLUTag cells [50], we investigated the ability of 1-CA-LPC to mobilize [Ca^2+^]_i_. Indeed, 1-CA-LPC was effective at stimulating [Ca^2+^]_i_ mobilization, and this effect was *diminished* by applied *antagonists*.

The highest cytotoxic effect on the MIN6 cells’ growth observed for conjugates of PC with sesquiterpenes 1-GERA-LPC and 1-FARA-LPC can be the effect of higher lipophilicity of these compounds in comparison to the lysophosphatidylcholines containing monoterpene acids 1-GA-LPC and 1-CA-LPC. However, the most significant factor determining the toxicity of 1-GERA-LPC and 1-FARA-LPC is probably the vinyl moiety in their structures which does not occur in the natural isoprenoid acids like geranic acid and citronellic acid. In conclusion, in pancreatic β-cells, 1-CA-LPC and 1-GA-LPC were potent at stimulating insulin secretion under high glucose concentration. This phenomenon is of crucial importance because the positive effect on insulin secretion must be relevant physiologically. This appears to be attributable to their ability to trigger intracellular calcium level elevation via four GPCRs, namely GPR40, GPR55, GPR119, and GPR120, involved in maintaining lipid and carbohydrate homeostasis [7]. Furthermore, 1-CA-LPC exerted efficacy in the stimulation of GLP-1 secretion in enteroendocrine L-cells targeting GPR55, GPR119, and GPR120.

Overall, modulating the activity of GPCRs represents one of the most important approaches in modern drug discovery. Targeting pancreas GPCRs (e.g., GPR40, GPR55, GPR119, GPR120) could regulate islet function and hormone secretions, ultimately controlling glucose homeostasis [7]. On the other hand, natural endogenous LPC controls critical biological pathways. LPC was first proposed as an endogenous ligand for GPR119, which resulted in the stimulation of GSIS [8]. Recent results indicate that GPR55 and GPR40 can also be activated by LPC [13]. In the studies presented here, our data demonstrate that insulin and GLP-1 release evoked by isoprenoid derivatives of LPC seemed to be GPR40-, GPR55-, GPR119- and GPR120-dependent. Notably, 1-GA-LPC and 1-CA-LPC appeared to be less toxic as compared to natural LPCs with fatty acid residues. Natural LPCs are cone-shaped molecules with hydrophobic tails and polar heads that can easily penetrate lipid bilayers and destroy plasma membrane integrity. When LPCs are used in concentrations close to the critical micelle concentration (CMC), the lipid bilayer may be ruptured or even completely dissolved. The length and saturation of the fatty acid determine the CMC that for 16:0 LPC is approximately about 7–10 [51]. 1-GA-LPC and 1-CA-LPC did not cause any toxicity up to 100 µM. Therefore, LPCs bearing a prenylated side chain (especially 1-CA-LPC) may serve as a new therapeutic approach in the therapy of diabetes. However, further research is required to validate this finding in vivo and determine the stability and bioavailability of isoprenoid LPCs.

## 4. Materials and Methods

### 4.1. Chemicals and Reagents 

Supplements, culture media, and phosphate-buffered saline (PBS; pH 7.4), PrestoBlue Cell Viability Reagent, CyQUANT Cell Proliferation Assay Kit, and DMSO were obtained from Life Technologies (Carlsbad, CA, USA). Propidium iodide, glucagon-like peptide 1 (GLP-1), ethanol, β-mercaptoethanol, amphotericin B, penicillin, and neomycin were purchased from Sigma-Aldrich (Saint Louis, MO, USA). Lysis Buffer was obtained from R&D Systems, Inc. (Minneapolis, MN, USA), Bradford Protein Assay from *Bio-Rad* (Hercules, CA, USA), Screen QuestTM Fluo-8 No Wash Calcium Assay Kit from AAT Bioquest, Inc. (Sunnyvale, CA, USA), Mouse Glucagon-Like Peptide 1 (GLP-1) ELISA kit from-BlueGene (Shanghai, China) detecting both active and inactive forms of GLP-1. Reagents for quantitative reverse-transcription PCR (CellAmp Direct RNA Prep Kit RT-PCR and One Step TB Green Ex Taq qRT-PCR Kit) were obtained from Takara Bio Inc. (Shiga, Japan).

Selective antagonists of GPR40 (DC260126, depicted as DC), GPR55 (CID16020046, depicted as CID), and GPR120 (AH7614, depicted as AH) were obtained from Tocris Bioscience (Ellisville, MO, USA). The GPR119 antagonist (C8) was kindly provided by Pfizer (Groton, CT, USA) [52]. Antagonists were formulated as 10 mM stock solutions in DMSO and used at 2 M working concentrations in cell culture studies [13].

Isoprenoid derivatives of lysophosphatidylcholine: 1′-[3,7-dimethyl-3-vinylocta-6-enyl]-2′-hydroxy-*sn*-glycero-3′-phosphocholine (1-GERA-LPC), 1-geranoyl-2-hydroxy-*sn*-glycero-3-phosphocholine (1-GA-LPC), 1-citroneloyl-2-hydroxy-*sn*-glycero-3-phosphocholine (1-CA-LPC), 1′-[3,7,11-trimethyl-3-vinyldodec-6,10-dienyl]-2′-hydroxy-*sn*-glycero-3′-phosphocholine (1-FARA-LPC), were originally synthesized at the Department of Chemistry, Wrocław University of Environmental and Life Sciences as described previously [39,40]. Free monoterpene acids: geranic acid (GA), cytronellic acid (CA) were purchased from Sigma Aldrich whereas 2,3-dihydro-3-vinylgeranic acid (GERA), and free sesquiterpene acid: (E)-3,7,11-trimethyl-3-vinyldodeca-6,10-dienoic acid (FARA), were synthesized according to the previously described procedure [39,40]. Solid compounds were solubilized in DMSO:EtOH solution (1:1) in 100 mM concentrations further diluted in PBS or cell culture media.

### 4.2. MIN6 Cell Culture 

The murine insulinoma MIN6 cells were generously donated by Prof. Peter Bergsten (Uppsala University, Uppsala, Sweden) with the permission of Dr. Jun-ichi Miyazaki (Division of Stem Cell Regulation Research, Osaka University, Osaka, Japan) [53]. The culture medium consisted of DMEM containing 4.5 g/L glucose, 10% fetal bovine serum, 50 μM β-mercaptoethanol, 200 mg/L penicillin, 50 mg/L neomycin, 2.5 mg/L amphotericin B. The culture was conducted at 37 °C in a humidified atmosphere of 5% CO_2_. The experiments were performed between passages 24–30.

### 4.3. GLUTag Cell Culture 

The murine GLUTag cells were kindly provided by Prof F. Urbano (University of Catania, Catania, Italy) with the permission of Prof. Daniel J. Drucker (University of Toronto, Toronto, ON, Canada) [54]. Cells were cultured in DMEM medium with 5.6 mM glucose, supplemented with 10% fetal bovine serum, 200 mg/L penicillin, 50 mg/L neomycin, 2.5 mg/L amphotericin B. The culture was conducted at 37 °C in a humidified atmosphere of 5% CO_2_. The experiments were performed between passages 22–28.

### 4.4. Cell Viability 

MIN6 and GLUTag cells were seeded onto 96-well plates in the amount of 4 × 10^4^ cells per well 48 h. Next, the respective culture media were modified to be serum-free and supplemented with the compounds (5 μM; 10 μM; 25 μM; 50 μM; 100 μM; 500 μM for MIN6 and 5 μM; 10 μM; 25 μM for GLUTag). The fluorescent signal at F530/620 nm was measured to determine cell viability after 24 or 48 h of exposure to the tested compounds using PrestoBlue Cell Viability Reagent according to the manufacturer’s instructions. Cell viability was also evaluated using CyQUANT^TM^ Cell Proliferation Assay Kit according to the manufacturer’s instructions. In this case, the concentrations of tested compounds and their respective solvent controls (DMSO:EtOH) were 5 μM, 10 μM, 25 μM. After 24 or 48 h of exposure to the tested compounds, cell viability was measured by measuring the fluorescent signal at F485/528 nm. The obtained fluorescence values in both tests were used to measure cell viability, which was expressed as a percentage of untreated control cells’ viability.

### 4.5. Glucose-Stimulated Insulin Secretion (GSIS) 

MIN6 cells were seeded in the amount of 2 × 10^5^ per well 48 h on 24-well plates. For the first 60 min, confluent cells were pre-incubated with a calcium buffer (25 mM HEPES, 125 mM NaCl, 6 mM KCl 1.2 mM MgCl_2_:6H_2_O, 1.3 mM CaCl_2_:2H_2_O, pH 7.4) supplemented with 2 mM glucose. Subsequently, cells were incubated in the new portions of the same buffer supplemented with LPCs or their corresponding acids at either 5, 10, or 25 μM working concentrations and/or GPCR antagonists (2 μM of DC, CID, C8, and AH) for 30 min. MIN6 cells were also stimulated with glucagon-like peptide (GLP-1) in which case 50 nM, 100 nM, 500 nM, 1 μM, or 5 μM concentrations were tested. The buffer supernatants were saved and the same cells were incubated for another 30 min with 20 mM glucose buffer supplemented with test compounds. After the collection of buffer samples from the high-glucose conditions, cells were lysed with lysis buffer and subjected to competitive ELISA [55]. The protein content of respective cell lysates was determined using the Bradford Protein Assay, and the amounts of secreted insulin were normalized.

### 4.6. Glucagon-Like Peptide 1 (GLP-1) Secretion

GLUTag cells were seeded on 24-well plates in the amount of 2 × 10^5^ per well 48 h. Confluent cells were pre-incubated for 60 min at fasting conditions (5.6 mM glucose DMEM). Subsequently, cells were incubated in the new portions of the serum-free medium supplemented with tested compounds at either 10 μM working concentrations and/or GPCR antagonists (2 μM of DC, CID, C8, and AH) for 120 min. The buffer supernatants were collected, and cells were lysed with lysis buffer were subjected for competitive ELISA, using the Mouse GLP-1 ELISA Kit according to the supplier’s protocol. Quantities of secreted GLP-1 were normalized to protein contents in respective cell lysates measured according to Bradford Protein Assay.

### 4.7. Intracellular Calcium ([Ca^2+^]_i_) Mobilization

MIN6 cells were seeded at a density of 4 × 10^4^ cells per well in 96-well plates for 24 h. According to the manufacturer’s protocol, the intracellular calcium concentration was measured using the Screen QuestTM Fluo-8 No Wash Calcium Assay Kit. The buffer supplemented with either 2 mM or 20 mM glucose was used instead of the MIN6 culture medium. To track potential membrane permeabilization caused by the investigated compounds, propidium iodide was applied at a final concentration of 1 g/mL [19]. Flux was measured after stimulation with chosen compound and/or GPCR antagonists. [Ca^2+^]_i_ mobilization and PI intercalation were monitored simultaneously by the change in fluorescence (excitation/emission = 485/528 nm and 530/620 nm, respectively). The fluorescence reads were compared to the results obtained after the cells were stimulated with the compound solvent (DMSO:EtOH).

In experiments on GLUTag cells the intracellular calcium concentration [Ca^2+^]_i_ was also evaluated. The experiment was performed analogically, however, performed in 5.6 mM glucose DMEM, corresponding to the conditions of the GLP-1 secretion experiment.

### 4.8. Statistical Analysis

The results are presented as means of 3–6 repeated experiments (3–6 biological repeats each) ± SEM. Groups of data passed normality distribution tests and were compared using either two-way ANOVA (cytotoxicity experiments and GSIS when insulin secretion was taken into account in low and high glucose conditions) or one-way ANOVA with Bonferroni post hoc test. *p* < 0.05 was considered as statistically significant. The results obtained after stimulation of the cell model with investigated compounds were compared to the respective solvents. The results obtained after stimulation of the cell model with investigated compounds were compared to the respective solvent (DMSO:EtOH), for high (*) and low glucose conditions (^, applicable in the case of GSIS and [Ca^2+^]_i_ mobilization experiments in MIN6 as well as all experiments performed in GLUTag cells). The statistically significant difference between the cell model treated with the compound of interest and a particular receptor antagonist simultaneously (#) was shown whenever GPCR-mediated activity was tested. GraphPad Prism v. 8.3 (GraphPad Software, La Jolla, CA, USA) was used to assess the statistical significance of the obtained data.

## Data Availability

The data presented in this study are available on request from the corresponding authors.
